# CRISPR-SWITCH (silent mutations with intention to create heterozygotes): a strategy for monoallelic genome editing and generation of a *Syt1*-D365E mouse model of Baker–Gordon syndrome

**DOI:** 10.3389/fgeed.2026.1833024

**Published:** 2026-07-10

**Authors:** Samantha Norris, Sai Goutham Reddy Yeddula, Elaine Su, Klancey Vandeloecht, Sandy Saunders, Yoko Wang, Carie Boychuk, W. David Arnold, Christian Lorson, Daniel J. Davis

**Affiliations:** University of Missouri, Columbia, MO, United States

**Keywords:** allele-specific targeting, Baker–Gordon syndrome, CRISPR-Cas9, CRISPR-SWITCH, forced heterozygosity, monoallelic genome editing, mouse model, synaptotagmin-1 (SYT1)

## Abstract

Precise control of allelic outcomes remains a major limitation of CRISPR-Cas9 genome editing, particularly for genes in which biallelic modification is lethal or confounds disease modeling. Here, we present CRISPR-SWITCH (Silent mutations With Intention To Create Heterozygotes), a genome engineering strategy that enables deliberate monoallelic editing by exploiting allele-specific CRISPR targeting. CRISPR-SWITCH operates through the initial introduction of a synonymous nucleotide substitution that creates a unique guide RNA recognition site, allowing subsequent selective editing of the engineered allele while preserving the wildtype copy. We applied CRISPR-SWITCH to generate a mouse model of Baker-Gordon syndrome, a dominant-negative neurodevelopmental disorder caused by pathogenic variants in synaptotagmin-1 (*SYT1*). Conventional CRISPR-Cas9 editing of the *Syt1* locus produced complex allelic outcomes characterized by biallelic editing and mosaicism, preventing reliable generation of the defined heterozygous genotype required for disease modeling. In contrast, CRISPR-SWITCH enforced heterozygosity by first introducing a synonymous Y364Y mutation and then selectively targeting this allele to install the pathogenic D365E variant. This approach produced viable *Syt1*-D365E mice with exclusive monoallelic genome editing, predictable preservation of a wildtype allele, and balanced (1:1) expression of mutant and wildtype transcripts. Together, these results demonstrate proof-of-principle that CRISPR-SWITCH can enforce heterozygosity at endogenous loci and enable the generation of viable mammalian models for dominant-negative and dosage-sensitive genetic disorders.

## Introduction

CRISPR-Cas9-mediated genome editing, particularly when coupled with homology-directed repair (HDR), has become one of the most widely used approaches for introducing precise genetic modifications *in vivo*. Its relative simplicity, flexibility, and cost effectiveness have enabled widespread adoption for the creation of transgenic and knock-in animal models (Doudna and Charpentier, 2014; Hsu et al., 2014). Despite these advantages, conventional CRISPR-Cas9 HDR strategies exhibit several well-documented limitations, including variable editing efficiency across loci and cell types, reduced efficiency for large insertions, and a strong tendency toward biallelic editing through various repair mechanisms when targeting essential genes in attempt to produce viable offspring (Gurumurthy and Lloyd, 2019; Hunt et al., 2023; Liao et al., 2024; Yeh et al., 2019; Zhang et al., 2022). Numerous technical refinements including: Easi-CRISPR, two-cell embryo microinjection, CRISPR-READI, and other AAV-mediated embryo delivery approaches have been developed to improve editing efficiency and precision (Quadros et al., 2017; Gu et al., 2018; Chen et al., 2019; Davis et al., 2023). However, these approaches are primarily optimized to maximize editing of both alleles rather than enabling controlled monoallelic modification.

Few alternative strategies to influence allelic editing outcomes have been explored. For example, Paquet et al. demonstrated that heterozygous outcomes can be enriched by increasing the distance between the Cas9-induced double-strand break and the desired mutation site, or by introducing equimolar mixtures of donor templates encoding distinct sequence modifications (Paquet et al., 2016). While these approaches can bias editing outcomes under certain conditions, they rely on multiple repair events and sequence-dependent parameters, and have primarily been validated *in vitro*. Moreover, these strategies do not prevent biallelic editing but instead shift the distribution of outcomes, limiting their utility for generating *in vivo* models in which strict monoallelic modification is required.

The lack of robust strategies for intentional monoallelic genome editing represents a critical gap in current CRISPR methodologies. For genes in which homozygous disruption is embryonic lethal, or where biallelic modification produces severe or confounding phenotypes, standard CRISPR approaches can prevent the generation of viable disease models. In these contexts, the ability to selectively edit only a single allelic copy, while preserving an intact wildtype allele, would substantially expand the range of genetically tractable targets and enable faithful modeling of dominant-negative and haploinsufficient disorders.

Allele-specific CRISPR-Cas9 targeting has been demonstrated in therapeutic contexts, where guide RNAs are designed to discriminate between mutant and wildtype alleles based on single-nucleotide differences or unique protospacer-adjacent motif (PAM) configurations (Christie et al., 2017; Wu et al., 2020; Bolduc et al., 2024; Cattaneo et al., 2024). However, this principle has not been systematically adapted as a model-generation strategy to deliberately enforce heterozygosity during genome editing. Here, we introduce CRISPR-SWITCH (Silent mutations With Intention To Create Heterozygotes), a two-step genome engineering approach that leverages allele-specific guide RNA recognition to enable precise, monoallelic editing at endogenous loci.

The CRISPR-SWITCH strategy operates by first introducing a synonymous (silent) mutation proximal to the desired pathogenic variant. This engineered nucleotide change creates a unique sequence that can be selectively recognized by a guide RNA without altering the overlapping protein coding sequence. In heterozygous embryos carrying one silent-modified allele and one wildtype allele, a guide RNA specific to the silent mutation directs Cas9-mediated cleavage exclusively to the modified allele. Subsequent HDR introduces the pathogenic variant solely on the modified allele, while the wildtype allele remains unedited. This approach effectively forces heterozygosity and avoids the unintended generation of homozygous or biallelic compound heterozygous mutations.

We applied CRISPR-SWITCH to generate a mouse model of Baker-Gordon syndrome, a rare neurodevelopmental disorder caused by dominant-negative mutations in the synaptotagmin-1 (*SYT1*) gene (Baker et al., 2018; Melland et al., 2022; Porto et al., 2024). *SYT1* encodes a synaptic vesicle protein that functions as a principal calcium sensor for synchronous neurotransmitter release. Pathogenic variants cluster within the C2A and C2B calcium-binding domains and disrupt synaptic vesicle dynamics, resulting in desynchronized neurotransmitter release and abnormal presynaptic regulation (Baker et al., 2018; Riggs et al., 2022). Clinically, affected individuals present with severe neurodevelopmental phenotypes including neonatal hypotonia, developmental delay, feeding difficulties, ocular abnormalities, behavioral disturbances, and sleep dysregulation (Baker et al., 2018; Melland et al., 2022).

Prior mouse studies have demonstrated that homozygous disruption of *Syt1* results in neonatal lethality, whereas heterozygous null animals are viable and phenotypically normal, indicating that preservation of at least one functional *Syt1* allele is sufficient for postnatal viability (Geppert et al., 1994). In contrast, heterozygous dominant-negative *SYT1* missense mutations produce severe, early-onset disease, presenting a major challenge for conventional CRISPR-based model generation approaches that can yield complex biallelic editing outcomes (Baker et al., 2018; Melland et al., 2022). To overcome this limitation, we developed CRISPR-SWITCH to introduce a patient-derived, disease-associated D365E (corresponding to human D366E) missense mutation into a single endogenous mouse *Syt1* locus. Human *SYT1* and mouse *Syt1* are highly conserved, sharing 98% amino acid sequence identity, with complete nucleotide conservation in the region flanking the D365E site. We first engineered a synonymous Y364Y silent mutation immediately upstream of the pathogenic site, then selectively targeted this modified allele using a guide RNA specific to the silent mutation. We hypothesized that this strategy would enable monoallelic introduction of the D365E mutation while preserving an unmodified wildtype allele. This forced heterozygosity would enable the successful generation of a viable mammalian model of Baker-Gordon syndrome that recapitulates the genetic architecture of the human disease.

More broadly, CRISPR-SWITCH provides a generalizable framework for monoallelic genome editing and expands the CRISPR toolkit for modeling dominant-negative, dosage-sensitive, and homozygous-lethal genetic conditions. By enabling precise control over allelic targeting, this approach facilitates the development of *in vivo* disease models that were previously difficult or impossible to generate using conventional CRISPR methodologies.

## Materials and methods

### Animals

All experimental procedures were approved by the University of Missouri Institutional Animal Care and Use Committee (IACUC) and were conducted in accordance with the National Institutes of Health Guide for the Care and Use of Laboratory Animals. Female (3 weeks of age) and male (8–10 weeks of age) C57BL/6J mice were obtained from The Jackson Laboratory (Strain: 000,664) and used for embryo production. Female and male CD1 mice (8–10 weeks of age) were obtained from Charles River Laboratories and used as surrogate dams and surgically vasectomized males (Strain: 022). Mice were housed in ventilated Thoren caging under standard conditions with *ad libitum* access to food and water and maintained on a 12 h light/12 h dark cycle.

### sgRNA and repair template design

The genomic sequence of the mouse synaptotagmin-1 (*Syt1*) gene was obtained from Ensembl (GRCm39). Single guide RNAs (sgRNAs) were designed using CRISPR RGEN Tools (Center for Genome Engineering, Korea) to minimize predicted off-target activity. Two sgRNAs were generated (listed in Supplementary Table 1): one targeting the wildtype *Syt1* exon 11 sequence and a second designed to selectively target the engineered synonymous Y364Y allele. Chemically synthesized sgRNAs were obtained from Synthego and modified with 2′-O-methyl analogs and 3′ phosphorothioate internucleotide linkages at the first three nucleotides of both the 5′ and 3′ terminal residues to enhance stability.

Two single-stranded oligodeoxynucleotide (ssODN) repair templates were designed (listed in Supplementary Table 1). The first ssODN introduced a synonymous thymine-to-cytosine substitution encoding the Y364Y silent mutation in exon 11 of *Syt1*. The second ssODN introduced a cytosine-to-adenine substitution encoding the disease-associated D365E missense mutation. Both ssODNs were complementary to the non-target strand and contained 100 bp homology arms flanking the desired mutation to facilitate HDR. Repair templates were synthesized as PAGE-purified ssODNs (Integrated DNA Technologies).

### CRISPR-Cas9 ribonucleoprotein assembly and zygote electroporation

CRISPR-Cas9 ribonucleoprotein (RNP) complexes were prepared immediately prior to electroporation. sgRNAs were combined with enhanced-specificity Cas9 protein (Sigma-Aldrich) and incubated at room temperature for 10 min to allow RNP formation. Following RNP assembly, the appropriate ssODN repair template was added to the mixture. Final electroporation concentrations were: sgRNA (100 ng/μL), Cas9 protein (100 ng/μL), ssODN (100 ng/μL).

Fertilized zygotes were collected from superovulated C57BL/6J females mated with C57BL/6J stud males using standard procedures. Zygotes were electroporated using a NEPA21 Super Electroporator (NepaGene) with a 1 mm gap glass slide electrode under the following conditions:

Poring pulse: 40 V, 3.5 ms duration, 50 ms interval, 10% decay rate, positive polarity (4 pulses).

Transfer pulse: 5 V, 50 ms duration, 50 ms interval, 40% decay rate, alternating polarity (5 pulses).

Following electroporation, embryos were cultured for 24 h and assessed for viability prior to surgical transfer into the ampullae of 0.5 dpc pseudopregnant CD1 females. For initial efficiency experiments embryos were left in culture until blastocyst formation (∼5 days).

### Genotyping and sanger sequencing

Genomic DNA was extracted from tail biopsies of offspring using a Qiagen DNeasy Blood and Tissue Kit according to the manufacturer’s instructions. PCR amplification of the *Syt1* exon 11 region was performed using EmeraldAmp HS Master Mix (Takara) and gene-specific primers (listed in Supplementary Table 1).

PCR cycling conditions were as follows:

95 °C for 3 min; 35 cycles of 95 °C for 30 s, 61 °C for 30 s, and 72 °C for 1 min; followed by a final extension at 72 °C for 7 min.

PCR products were purified using a NucleoSpin Gel and PCR Clean-Up kit (Macherey-Nagel), quantified using a NanoDrop 8,000 spectrophotometer, and submitted to the University of Missouri DNA Core Facility for Sanger sequencing. Sequencing chromatograms were analyzed to confirm successful incorporation of the Y364Y silent mutation or the D365E missense mutation.

Sanger sequencing chromatograms were analyzed using SeqScreener (Thermo Fisher Scientific) to determine editing outcomes and indel frequency. Monoallelic editing was defined by retention of a detectable wildtype *Syt1* sequence signal representing approximately one allele, together with evidence of editing affecting the Y364Y-targeted allele in the CRISPR-SWITCH approach.

### Bulk RNA sequencing

Cortex samples were collected from brains of postnatal day 1 (P1) CRISPR-SWITCH *Syt1*-D365E mutant mice and age-matched wildtype C57BL/6J controls. RNA was extracted using a Qiagen RNeasy Kit according to the manufacturer’s instructions and submitted to Azenta for bulk RNA sequencing.

Allelic expression analysis was performed by quantifying total *Syt1* reads and distinguishing wildtype *versus* D365E mutant transcripts based on the engineered nucleotide substitution. The proportion of wildtype and mutant *Syt1* expression was calculated as a percentage of total *Syt1* reads.

### Statistical analysis

Statistical analyses were performed using GraphPad Prism software. Allelic nucleotide rate differences between *Syt1*-D365E heterozygous mice and *Syt1*-WT controls were analyzed using a two-way analysis of variance (ANOVA) with genotype and allele (mutant *versus* wildtype) as independent variables, followed by Bonferroni’s multiple comparisons test. Data points represent individual animals. To evaluate inheritance of the *Syt1*-D365E allele, observed genotype frequencies from *Syt1*-D365E heterozygous × wildtype crosses were compared to the expected Mendelian 1:1 ratio using an unpaired t-test. Quantification of neonatal milk spot scores in wildtype and *Syt1*-D365E heterozygous pups was analyzed by statistical analysis performed on 38 offspring using nonparametric Mann-Whitney U two tailed t-test. A Fisher’s exact test using the number of mutant and wildtype neonates found dead versus alive determined a significant difference in mortality based on genotype. Statistical significance for all tests was defined as p < 0.05.

## Results

### Conventional CRISPR-Cas9 editing produces predominantly biallelic and mosaic *Syt1* editing outcomes

**FIGURE 1:**
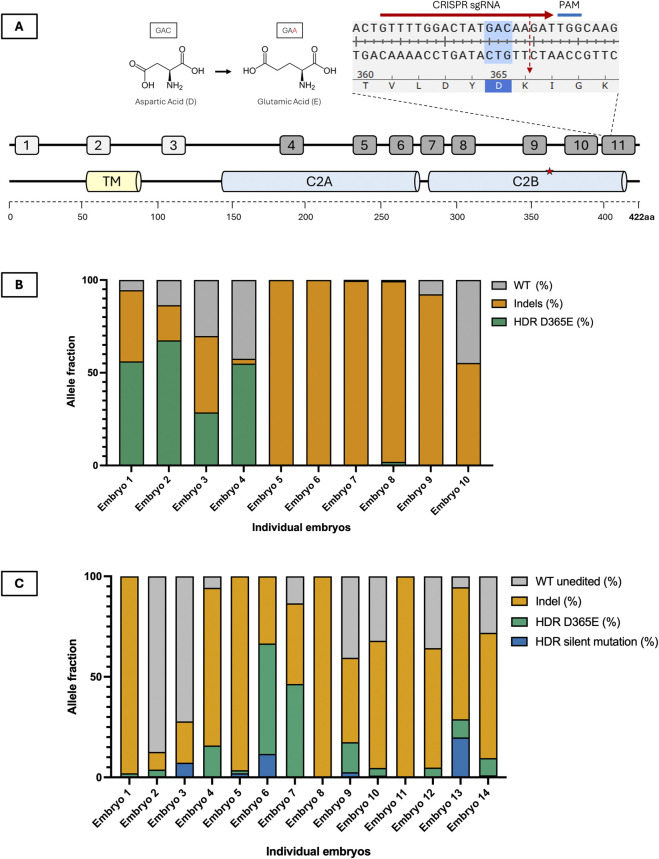
Conventional CRISPR-Cas9 editing produces biallelic and mosaic *Syt1* editing outcomes. **(A)** Schematic illustrating the mouse synaptotagmin-1 (*Syt1*) locus and the desired D365E single nucleotide substitution, which results in an aspartic acid (D) to glutamic acid (E) amino acid change within the calcium-binding C2B domain of synaptotagmin-1. The CRISPR sgRNA is indicated by the red arrow, the predicted Cas9 cleavage site by the dashed red arrow, the protospacer adjacent motif (PAM) sequence by the blue line, and location within protein domain by the red star. **(B)** Distribution of editing outcomes following electroporation of C57BL/6J zygotes with CRISPR-Cas9 RNP complexes and a D365E ssODN donor template. Embryos were cultured to the blastocyst stage and analyzed by Sanger sequencing (n = 10). Five embryos (50%) contained evidence of HDR-mediated incorporation of the D365E mutation. Embryos exhibited evidence of biallelic editing, and multiple edited alleles were frequently detected within individual embryos, consistent with mosaic repair events during early embryonic development. **(C)** Distribution of editing outcomes following electroporation of C57BL/6J zygotes with CRISPR-Cas9 RNP complexes and an equimolar mixture of D365E and Y364Y ssODN donor templates. Embryos were cultured to the blastocyst stage and analyzed by Sanger sequencing (n = 14). Four embryos (29%) contained evidence of both D365E and Y364Y edits. Despite incorporation of both donor-template sequences, embryos exhibited substantial biallelic editing and allelic mosaicism, indicating that mixed-donor strategies did not reliably generate defined monoallelic *Syt1*-D365E editing outcomes.

To determine whether conventional CRISPR-Cas9 HDR approaches could generate a viable heterozygous mouse model of Baker-Gordon syndrome, we selected the disease-associated *SYT1*-D366E missense mutation, which corresponds to D365E in the mouse *Syt1* gene. This mutation results from a cytosine-to-adenine substitution and produces an aspartic acid-to-glutamic acid amino acid change within the C2B calcium-binding domain of *Syt1* (Figure 1A). A guide RNA was designed to target exon 11 of *Syt1* adjacent to the D365E site, and a ssODN donor template was generated to introduce the desired mutation via HDR.

To evaluate editing outcomes, fertilized C57BL/6J zygotes were electroporated with CRISPR-Cas9 RNP complexes and the D365E donor template and cultured to the blastocyst stage prior to genotyping. Sequence analysis of individual blastocysts (n = 10) demonstrated successful incorporation of the D365E mutation in 5 embryos (50%) (Figure 1B). However, all embryos exhibited evidence of biallelic editing, with little wildtype sequence retained. Multiple edited alleles were frequently observed within individual embryos, consistent with mosaic editing arising from independent repair events during early embryonic development.

In an attempt to produce heterozygous embryos containing a functional *Syt1* allele, we next evaluated an alternative strategy based on simultaneous delivery of two donor templates, one encoding the D365E pathogenic variant and the other encoding the synonymous Y364Y mutation. Embryos electroporated with CRISPR-Cas9 RNP complexes and both donor templates were cultured and analyzed at the blastocyst stage (n = 14). Four embryos (29%) contained evidence of both the D365E and Y364Y edits (Figure 1C), indicating that dual-donor HDR can generate embryos carrying both desired sequence modifications. However, these embryos also showed extensive allelic mosaicism and mostly biallelic editing, with multiple edited alleles present within individual embryos. Because founder generation requires recovery of viable animals with a stable, transmissible genotype, the presence of these edits in blastocyst-stage embryos was not sufficient to establish that this approach would reliably produce usable D365E/Y364Y founders. Consistent with this concern, attempted founder generation using the dual-donor strategy did not yield viable pups for line establishment. This founder-generation attempt included 16 embryo transfers, with each surrogate female receiving 18–24 embryos by bilateral surgical transfer. These findings indicate that conventional CRISPR-Cas9 editing approaches, including multiplex donor-template strategies intended to bias heterozygous outcomes, produced complex allelic outcomes that were not well suited for generating stable *Syt1*-D365E founders with one functional *Syt1* allele. Collectively, these results motivated development of the CRISPR-SWITCH strategy to improve control over allelic outcomes during monoallelic genome editing.

### Generation of a synonymous C57BL/6J-*Syt1* ^Y364Y^ (*Syt1*-Y364Y) silent mutation line

To establish an allele-specific targeting site for CRISPR-SWITCH, we first introduced a synonymous Y364Y mutation immediately upstream of the desired D365E pathogenic variant. The Y364Y mutation consists of a thymine-to-cytosine substitution that preserves the encoded tyrosine residue while creating a unique nucleotide sequence that can subsequently be recognized by an allele-specific sgRNA.

**FIGURE 2:**
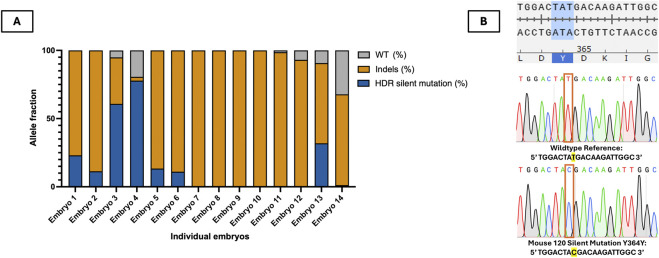
Generation of a synonymous *Syt1*-Y364Y founder line for CRISPR-SWITCH. **(A)** Distribution of editing outcomes following electroporation of C57BL/6J zygotes with CRISPR-Cas9 RNP complexes and a Y364Y ssODN donor template. Embryos were cultured to the blastocyst stage and analyzed by Sanger sequencing (n = 14). Seven embryos (50%) contained evidence of HDR-mediated incorporation of the Y364Y mutation. Similar to conventional D365E targeting, embryos frequently exhibited biallelic editing, INDEL formation, and multiple edited alleles consistent with mosaic repair events occurring during early embryonic development. **(B)** Representative Sanger sequencing chromatograms from a wildtype C57BL/6J mouse and founder animal #120 carrying the synonymous Y364Y mutation. The engineered thymine-to-cytosine substitution (highlighted) preserves the encoded tyrosine residue while creating a unique nucleotide sequence for allele-specific targeting. Founder #120 was used to establish the C57BL/6J-*Syt1* ^Y364Y^ (*Syt1*-Y364Y) line utilized for subsequent CRISPR-SWITCH experiments.

A CRISPR-Cas9 RNP complex and ssODN donor template were designed to introduce the Y364Y mutation through HDR. To characterize editing outcomes, electroporated embryos were cultured to the blastocyst stage and analyzed by Sanger sequencing (n = 14). Seven of fourteen embryos (50%) contained evidence of HDR-mediated incorporation of the Y364Y mutation. Similar to the editing outcomes observed with conventional D365E targeting, embryos frequently exhibited multiple edited alleles, including biallelic modification and indel formation, consistent with mosaic repair events occurring during early embryonic development (Figure 2A).

Because the Y364Y substitution does not alter the encoded amino acid sequence, biallelic incorporation of the mutation was not expected to affect viability. Founder screening following Y364Y targeting identified four offspring with distinct editing outcomes: one homozygous Y364Y HDR founder (#120), two mosaic animals containing evidence of the Y364Y HDR allele together with additional indel-containing alleles, and one unedited wildtype animal (Supplementary Figure 1). Sanger sequencing confirmed successful introduction of the synonymous nucleotide substitution in founder #120, and the animal exhibited normal viability despite modification of both *Syt1* alleles (Figure 2B).

Although either heterozygous or homozygous Y364Y founders could theoretically be used for CRISPR-SWITCH retargeting, founder #120 was selected because it carried two copies of the silent Y364Y allele without altering the encoded protein sequence and could be bred to establish a stable line. Establishing the Y364Y allele as a homozygous line provided a practical advantage for the second editing step: crossing homozygous Y364Y animals to wildtype C57BL/6J mice generated embryos uniformly heterozygous for the silent allele, ensuring that every embryo contained one engineered target allele and one wildtype allele prior to CRISPR-SWITCH editing. Subsequent breeding established a stable C57BL/6J-*Syt1* ^Y364Y^ (Syt1-Y364Y) line for use in CRISPR-SWITCH-mediated allele-specific targeting.

### CRISPR-SWITCH enables efficient monoallelic introduction of the *Syt1*-D365E mutation

**FIGURE 3:**
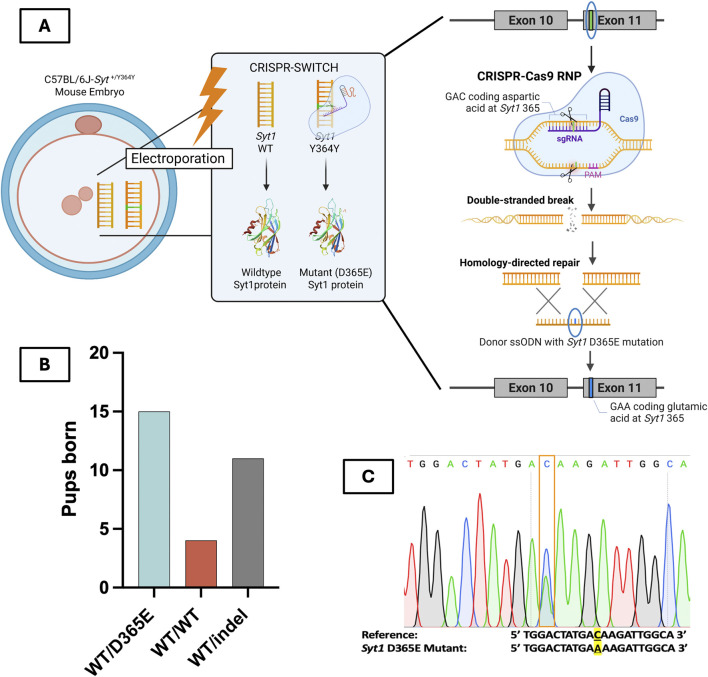
CRISPR-SWITCH enables efficient monoallelic introduction of the *Syt1*-D365E mutation. **(A)** Schematic illustrating the CRISPR-SWITCH strategy. Embryos heterozygous for the synonymous *Syt1*-Y364Y allele were generated by crossing homozygous *Syt1*-Y364Y mice with C57BL/6J wildtype mice. CRISPR-Cas9 reagents and a D365E ssODN donor template were then introduced to selectively target the Y364Y-modified allele and introduce a cytosine-to-adenine substitution encoding the D365E missense mutation via HDR, while preserving the unmodified wildtype allele. **(B)** Distribution of genotypes among offspring generated using CRISPR-SWITCH (n = 30). Fifteen animals (50%) carried the desired *Syt1*-D365E allele generated through HDR using the supplied ssODN donor template. Four animals (13%) retained only wildtype sequence at the targeted locus, consistent with repair using the homologous wildtype chromosome as a repair template. Eleven animals (37%) contained INDELs at the targeted allele resulting from alternative double-strand break repair pathways. Importantly, 100% of animals retained one unmodified wildtype *Syt1* allele, consistent with successful monoallelic targeting. **(C)** Representative Sanger sequencing chromatograms from a CRISPR-SWITCH founder demonstrating monoallelic incorporation of the *Syt1*-D365E mutation while preserving the wildtype allele. The D365E nucleotide substitution is highlighted.

To selectively introduce the pathogenic D365E mutation while preserving one wildtype allele, homozygous *Syt1*-Y364Y mice were crossed with C57BL/6J wildtype mice to generate embryos heterozygous for the silent mutation. A CRISPR-Cas9 RNP complex was then designed to selectively target the Y364Y-modified allele, together with an ssODN donor encoding the D365E mutation (Figure 3A).

Genotyping of resultant offspring by Sanger sequencing demonstrated efficient allele-specific editing and a marked improvement in control of allelic editing outcomes. Of the total neonates analyzed (n = 30), 100% retained one unmodified wildtype *Syt1* allele, consistent with successful monoallelic targeting. Among these animals, 15 (50%) carried the correctly edited *Syt1*-D365E allele generated through HDR, while 11 (37%) contained INDELs at the targeted allele resulting from alternative double-strand break repair pathways (Figure 3B).

Notably, four animals (13%) harbored only wildtype *Syt1* alleles despite successful targeting of the silent mutation locus. This outcome is consistent with repair of the targeted chromosome using the homologous wildtype allele as a repair template rather than the supplied ssODN donor. Such inter-homolog repair events have previously been reported in mammalian embryos and can result in gene conversion that restores the wildtype sequence at the targeted locus (Wilde et al., 2021; Zhang et al., 2022).

Importantly, unlike the conventional editing approaches described in Figures 1, 2, all edited animals generated through CRISPR-SWITCH retained a single wildtype *Syt1* allele, demonstrating highly controlled monoallelic targeting of the locus. Two founders carrying monoallelic *Syt1*-D365E edits were selected for line establishment (Figure 3C). Both founders successfully transmitted the D365E allele through the germline following backcrossing to C57BL/6J mice, establishing the C57BL/6J-*Syt1* ^D365E^ (*Syt1*-D365E) line.

### Allele-specific RNA expression confirms forced heterozygosity in *Syt1*-D365E mice

To confirm that CRISPR-SWITCH-mediated editing produced balanced expression of both *Syt1* alleles, bulk RNA sequencing was performed on brain tissue collected from postnatal day 1 (P1) *Syt1*-D365E heterozygous mice (n = 3) and age-matched wildtype controls (n = 3). *Syt1* is robustly expressed in the developing brain at this stage, allowing quantitative assessment of allele-specific transcript abundance.

**FIGURE 4:**
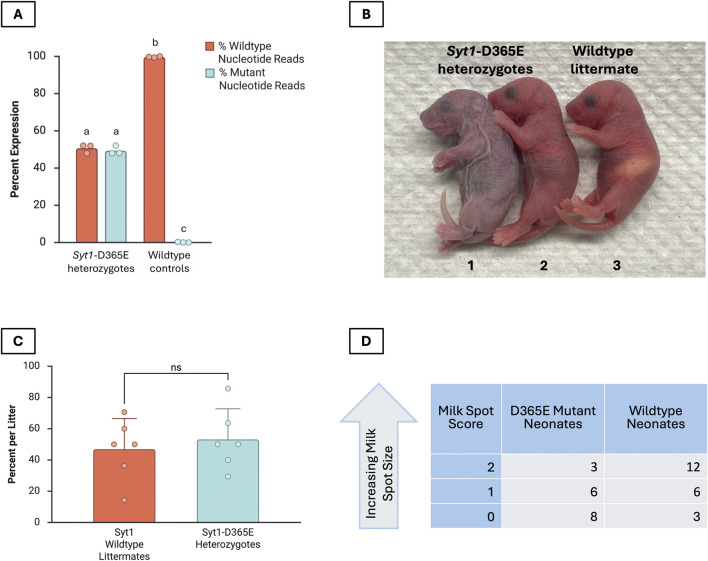
*Syt1*-D365E mice exhibit balanced allelic expression, normal Mendelian inheritance, and early postnatal feeding deficits. **(A)** Allele-specific expression analysis of *Syt1* transcripts from postnatal day 1 (P1) brain tissue. Bulk RNA sequencing demonstrated approximately equal expression of wildtype and D365E mutant transcripts in *Syt1*-D365E heterozygous mice (n = 3), whereas wildtype controls (n = 3) exhibited exclusively wildtype transcript expression. Data are presented as the percentage of total *Syt1* reads corresponding to each allele. Statistical significance using a two-way ANOVA followed by Bonferroni’s multiple comparisons test is represented by labels a, b, c where a different letter indicates statistical significance with a p < 0.0001 (i.e., a-a = ns; a-b, b-c, and a-c < 0.0001). **(B)** Representative images of neonatal pups. Two *Syt1*-D365E heterozygous pups are shown, including one pup lacking a visible milk spot and one pup that died during the neonatal period. A wildtype littermate exhibiting a prominent milk spot indicative of successful nursing is shown for comparison. **(C)** Genotype distribution of offspring obtained from *Syt1*-D365E heterozygous × wildtype crosses. Wildtype and heterozygous pups were recovered at approximately equal frequencies, consistent with the expected Mendelian 1:1 inheritance ratio. No significant difference was noted in inheritance based on genotype using an unpaired t-test. **(D)** Quantification of neonatal milk spot scores in wildtype and *Syt1*-D365E heterozygous pups. Milk spots were scored using a three-tier classification system: score 0, no visible milk spot; score 1, small milk spot; score 3, large milk spot. Heterozygous pups exhibited an increased frequency of absent milk spots and a reduced frequency of large milk spots compared with wildtype littermates, consistent with impaired nursing behavior and early-life feeding deficits. Statistical analysis performed on 38 offspring using nonparametric Mann-Whitney U two tailed t-test. p = 0.008.

Genome-wide expression analysis revealed no major transcriptional alterations in mutant animals relative to wildtype controls, although additional analyses and increased sample sizes would be needed to fully evaluate (Supplementary Figure 2). Targeted analysis of *Syt1* transcripts spanning the D365E locus identified both wildtype and mutant alleles in heterozygous animals, with expression occurring at an approximately 1:1 ratio. Quantification of sequencing reads demonstrated that *Syt1*-D365E mice expressed an average of 49.34% mutant and 50.66% wildtype transcripts, whereas wildtype controls exhibited 99.7% wildtype and 0.3% mutant reads, consistent with background sequencing noise (Figure 4A; Supplementary Figure 3).

These findings confirm that CRISPR-SWITCH successfully generated a genetically and transcriptionally heterozygous *Syt1*-D365E model and that the mutant allele is expressed at levels comparable to the endogenous wildtype allele.

### *Syt1*-D365E heterozygous mice exhibit postnatal feeding deficits and increased neonatal mortality

To determine whether the *Syt1*-D365E mutation affected viability, offspring generated from *Syt1*-D365E heterozygous × wildtype crosses were genotyped at birth. Heterozygous and wildtype pups were recovered at an approximately 1:1 ratio (Figure 4C), consistent with expected Mendelian inheritance. A chi-square test detected no significant deviation from the expected 1:1 distribution, indicating that monoallelic expression of the D365E mutation does not result in embryonic lethality.

Although heterozygous animals were born at expected frequencies, many displayed evidence of impaired postnatal feeding. Visual inspection of neonatal pups revealed reduced or absent milk spots in a subset of *Syt1*-D365E animals compared to wildtype littermates (Figure 4B). To quantify this phenotype, milk spot size was scored at birth using a three-tier classification system. Complete absence of a visible milk spot (score 0) was observed in eight heterozygous pups compared to three wildtype littermates, while large milk spots indicative of successful nursing (score 2) were observed in only three heterozygous pups compared to twelve wildtype animals (Figure 4D). Intermediate milk spot scores (score 1) occurred at similar frequencies between genotypes.

Consistent with these feeding deficits, heterozygous *Syt1*-D365E animals exhibited increased neonatal mortality (Table 1). Mortality occurred during the early postnatal period despite the absence of gross anatomical abnormalities at birth. Because heterozygous animals were recovered at expected Mendelian frequencies and showed no evidence of prenatal loss, these findings indicate that the observed mortality occurs during the postnatal period and is likely associated with impaired feeding and failure-to-thrive phenotypes rather than embryonic developmental defects. Similar survival outcomes were observed in reciprocal breeding schemes, indicating that the phenotype is attributable to pup genotype rather than maternal effects.

**TABLE 1:** Offspring from *Syt1*-D365E founder mice were monitored after birth. All weaned offspring and any neonatal pups that were found dead were genetically characterized by heat melt analysis or sanger sequencing. The percent mortality for each litter was determined by dividing the number of neonatal pups found dead by the total number of pups confirmed for that genotypic makeup. The *Syt1*-D365E mutant offspring exhibited increased neonatal lethality across litters shown by the higher percent mortality compared to their wildtype litter mates.

​	D365E mutant neonates		Wildtype neonates	
Litter	# pups found deceased	% mortality	# pups found deceased	% mortality
1	1/2	50%	0/13	0%
2	2/2	100%	0/9	0%
3	0/2	0%	0/5	0%
4	0/4	0%	0/4	0%
5	1/1	100%	0/7	0%
6	2/3	66%	0/4	0%
7	4/4	100%	0/1	0%
8	0/2	0%	0/4	0%
9	0/6	0%	0/1	0%
10	0/3	0%	0/4	0%
11	2/4	50%	0/6	0%
12	0/4	0%	1/4	25%

Collectively, these data demonstrate that monoallelic expression of the *Syt1*-D365E mutation produces an early-life phenotype characterized by impaired nursing behavior, failure to thrive, and increased neonatal mortality, consistent with the severe neonatal manifestations reported in individuals with Baker-Gordon syndrome.

## Discussion

In this study, we developed CRISPR-SWITCH, a genome engineering strategy that enables deliberate monoallelic editing through allele-specific CRISPR-Cas9 targeting. Using this approach, we generated a *Syt1*-D365E mouse model of Baker-Gordon syndrome while maintaining an unmodified wildtype allele. Conventional editing approaches produced complex repair outcomes characterized by biallelic editing, allelic mosaicism, and multiple competing repair products, making it difficult to obtain the precise heterozygous genotype required for disease modeling. CRISPR-SWITCH addressed this challenge by providing predictable control over allelic editing outcomes through sequential allele-specific targeting.

Although HDR enables precise sequence changes, it frequently exhibits low efficiency and competes with non-homologous end joining (NHEJ) and other DNA repair mechnisms, particularly in early embryos and *in vivo* contexts. HDR efficiency can be limited in non-dividing cells, and NHEJ often predominates, resulting in indels and unwanted biallelic outcomes even when precise edits are intended (Liao et al., 2024). Our initial attempts to generate founder animals by direct D365E targeting did not yield viable founders for line establishment. Although the underlying genotypes from these attempts could not be resolved, embryo-stage analysis demonstrated that direct D365E targeting produced extensive biallelic and mosaic editing outcomes. These findings indicate that conventional CRISPR-Cas9 editing was poorly suited for recovering stable *Syt1*-D365E founders with one functional *Syt1* allele. Double-strand break repair frequently results in a mixture of HDR and NHEJ events across both alleles. While low HDR efficiency and competing NHEJ are widely recognized as primary limitations of CRISPR editing, these factors can lead to unintended biallelic modification in cases where editing does occur, posing a particular challenge for modeling genes in which homozygous mutation is lethal or confounding. These inherent challenges have spurred development of alternative genome-editing strategies but have largely focused on maximizing editing efficiency rather than controlling allelic outcomes.

CRISPR-SWITCH directly addresses this gap by converting a synonymous mutation into an allele-specific targeting handle, shifting the editing paradigm from probabilistic heterozygosity to enforced monoallelic modification. Allele-specific CRISPR targeting has been explored primarily in therapeutic contexts, where guide RNAs are designed to discriminate between alleles based on sequence differences to selectively modify mutant copies while sparing wildtype alleles. Prior studies have demonstrated the feasibility of allele-targeted CRISPR guides that preferentially modify one allele over another, including successful targeting of the *COL6A1* dominant-negative mutation in cultured cells (Bolduc et al., 2024). Such strategies underscore the potential of single-nucleotide differences to confer allele specificity, but these approaches generally depend on naturally occurring variants and are typically applied in therapeutic or *ex vivo* settings. CRISPR-SWITCH extends the allele-specific concept to model generation, enabling intentional creation of an allele-specific sequence that can be targeted reproducibly to enforce heterozygosity.

Some alternative strategies for influencing allelic outcomes have been described, including the use of mixed donor templates or manipulation of the distance between the Cas9-induced double-strand break and the desired mutation site (Paquet et al., 2016). These approaches can bias editing outcomes toward heterozygosity under defined conditions, particularly validated in cultured cells. Consistent with these findings, we observed that co-delivery of equimolar donor templates encoding the D365E and Y364Y mutations was capable of generating heterozygous alleles *in vivo*. However, this approach was less efficient and less predictable than CRISPR-SWITCH, likely due to the requirement for two independent HDR events occurring within the same embryo. In contrast, CRISPR-SWITCH decouples these events into sequential steps, thereby increasing control over allelic targeting and improving reproducibility of monoallelic outcomes. Additionally, strategies based on double-strand break positioning are inherently sequence-dependent and do not fully eliminate the risk of biallelic modification. Together, these comparisons highlight that while existing approaches can bias editing outcomes, CRISPR-SWITCH provides a more robust and predictable framework for enforcing heterozygosity *in vivo*.

A key strength of CRISPR-SWITCH is its ability to enforce heterozygosity not only at the genomic level but also at the level of gene expression. Bulk RNA sequencing of *Syt1*-D365E mice revealed near-equal expression of mutant and wildtype transcripts, confirming that monoallelic editing translated into balanced allelic output. This is important for modeling dominant-negative disorders, where gene dosage and relative expression levels critically influence disease severity and phenotypic presentation. Balanced allelic expression also supports the absence of cryptic allele skipping or compensation mechanisms that could bias transcript output.

Application of CRISPR-SWITCH enabled the generation of a viable mouse model that recapitulates key early-life features associated with Baker-Gordon syndrome. Synaptotagmin-1 is a principal calcium sensor for fast synchronous neurotransmitter release, and dominant-negative mutations in the *SYT1* gene disrupt synaptic vesicle function and neurodevelopment. Prior studies have demonstrated that pathogenic variants within the C2 domains impair calcium-dependent vesicle fusion and alter presynaptic regulation (Yoshihara et al., 2010; van Boven et al., 2024). In our *Syt1*-D365E mice, we observed early-life failure to thrive and increased neonatal lethality, phenotypes that are consistent with the severe neonatal hypotonia and feeding difficulties reported in affected individuals. Additional phenotyping is part of an ongoing manuscript in development. These findings support the biological relevance of the model further illustrating that CRISPR-mediated monoallelic introduction of pathogenic mutations can produce robust *in vivo* models for dominant-negative disorders, extending beyond conventional knockouts or transgenic overexpression systems.

CRISPR-SWITCH offers several advantages over conventional CRISPR strategies, including predictable allelic outcomes, preservation of an unmodified wildtype allele, and compatibility with existing genome editing delivery systems. However, the requirement for a two-step editing process introduces additional time and breeding steps. In addition, allele-specific targeting based on single-nucleotide differences can be difficult to predict from guide RNA design alone, and empirical validation of allele-specific guide RNA activity would strengthen future applications of CRISPR-SWITCH prior to embryo editing. Although we observed preservation of the wildtype allele in all CRISPR-SWITCH edited animals analyzed in this study, comprehensive off-target and allele-specific cleavage profiling would further strengthen confidence in guide specificity and genomic integrity.

CRISPR-SWITCH is conceptually applicable to genes for which homozygous mutation is lethal or where controlled monoallelic modification is required. However, as this study demonstrates application at a single locus (*Syt1*), additional validation across diverse genomic targets will be important to establish the broader robustness and generalizability of this approach.

In conclusion, CRISPR-SWITCH expands the CRISPR genome editing toolkit by enabling deliberate and reproducible monoallelic modification through allele-specific targeting of engineered silent mutations. By overcoming a fundamental limitation of conventional CRISPR-Cas9 approaches, this method provides a practical and reproducible strategy for generating monoallelic disease models in cases where conventional CRISPR approaches yield undesirable biallelic outcomes, particularly for dominant-negative or dosage-sensitive mutations.

## Data Availability

The datasets presented in this study can be found in online repositories. The names of the repository/repositories and accession number(s) can be found in the article/Supplementary Material.
