# Supplementary figures and images for: CRISPR-SWITCH (silent mutations with intention to create heterozygotes): a strategy for monoallelic genome editing and generation of a Syt1-D365E mouse model of Baker–Gordon syndrome
Source: Front Genome Ed. 2026 Jul 10;8:1833024. doi: 10.3389/fgeed.2026.1833024 (PMC13397936; doi:10.3389/fgeed.2026.1833024)

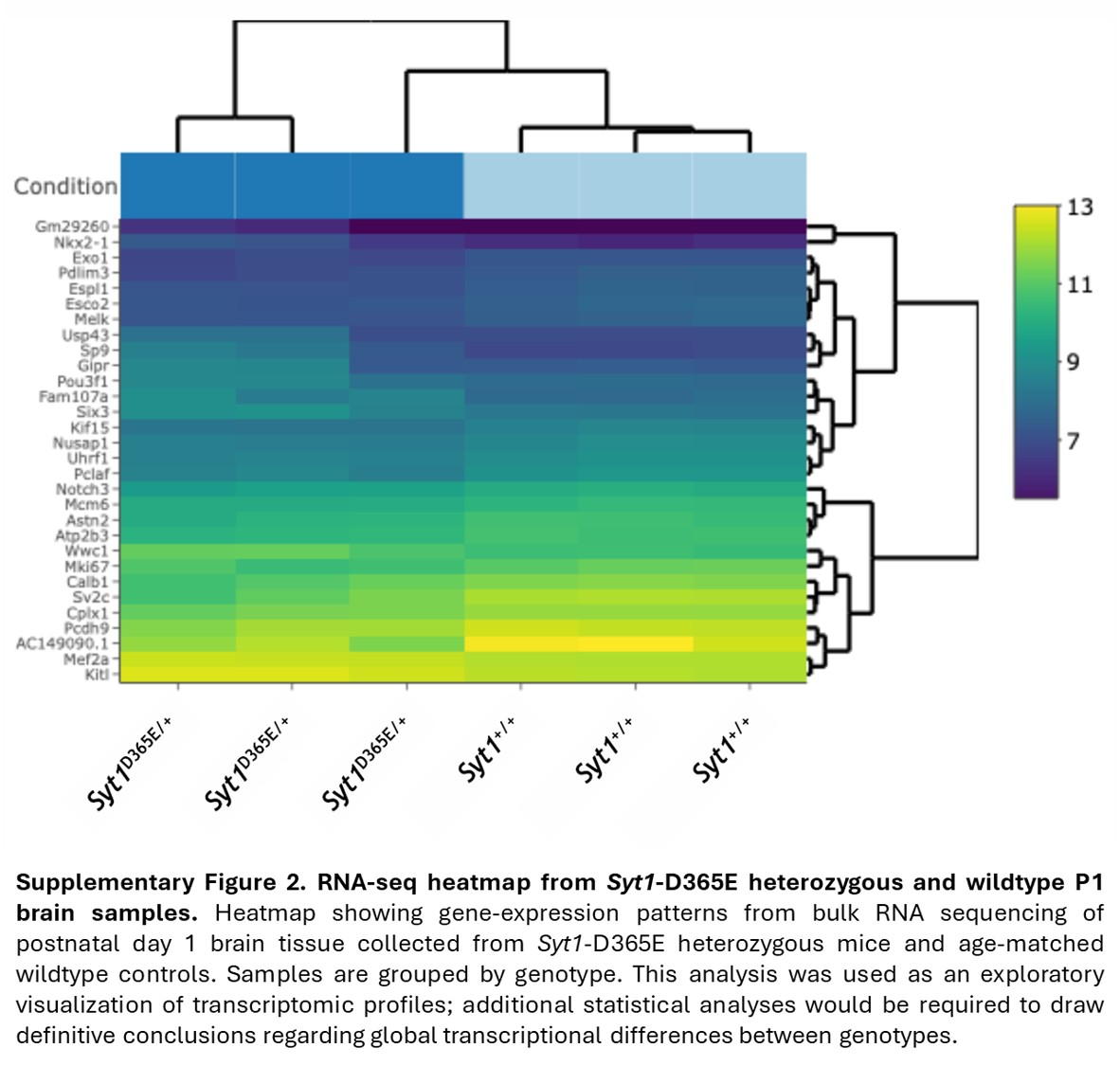

Supplement: Supplementary file 1 [file Image3.jpeg]

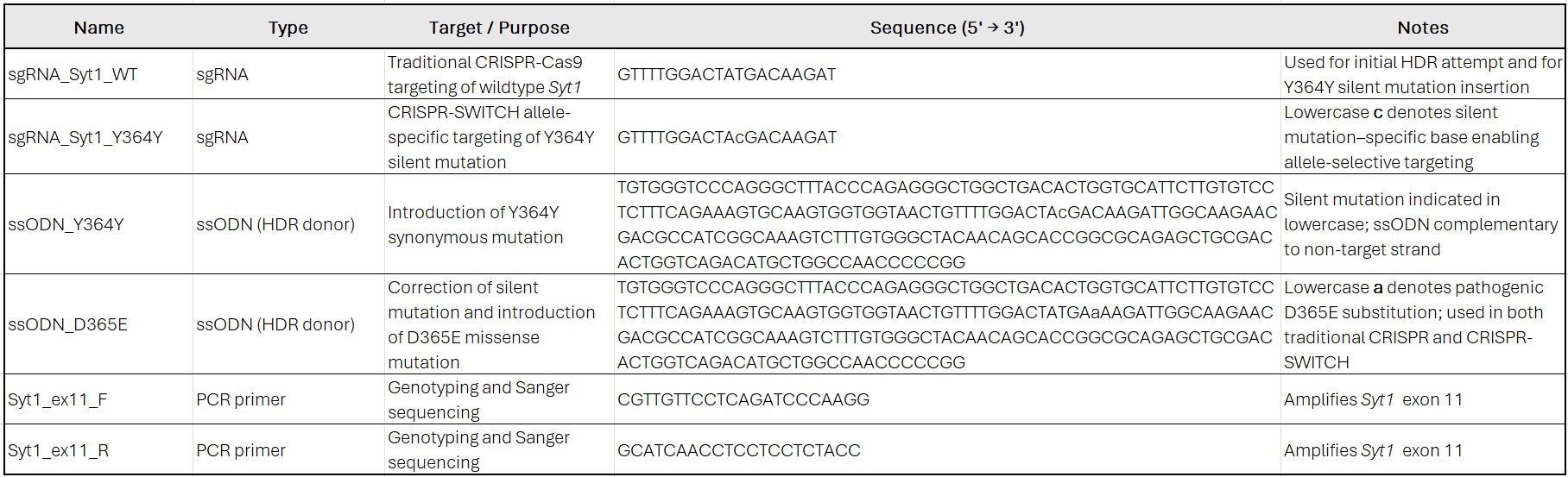

Supplement: Supplementary file 2 [file Image1.jpeg]

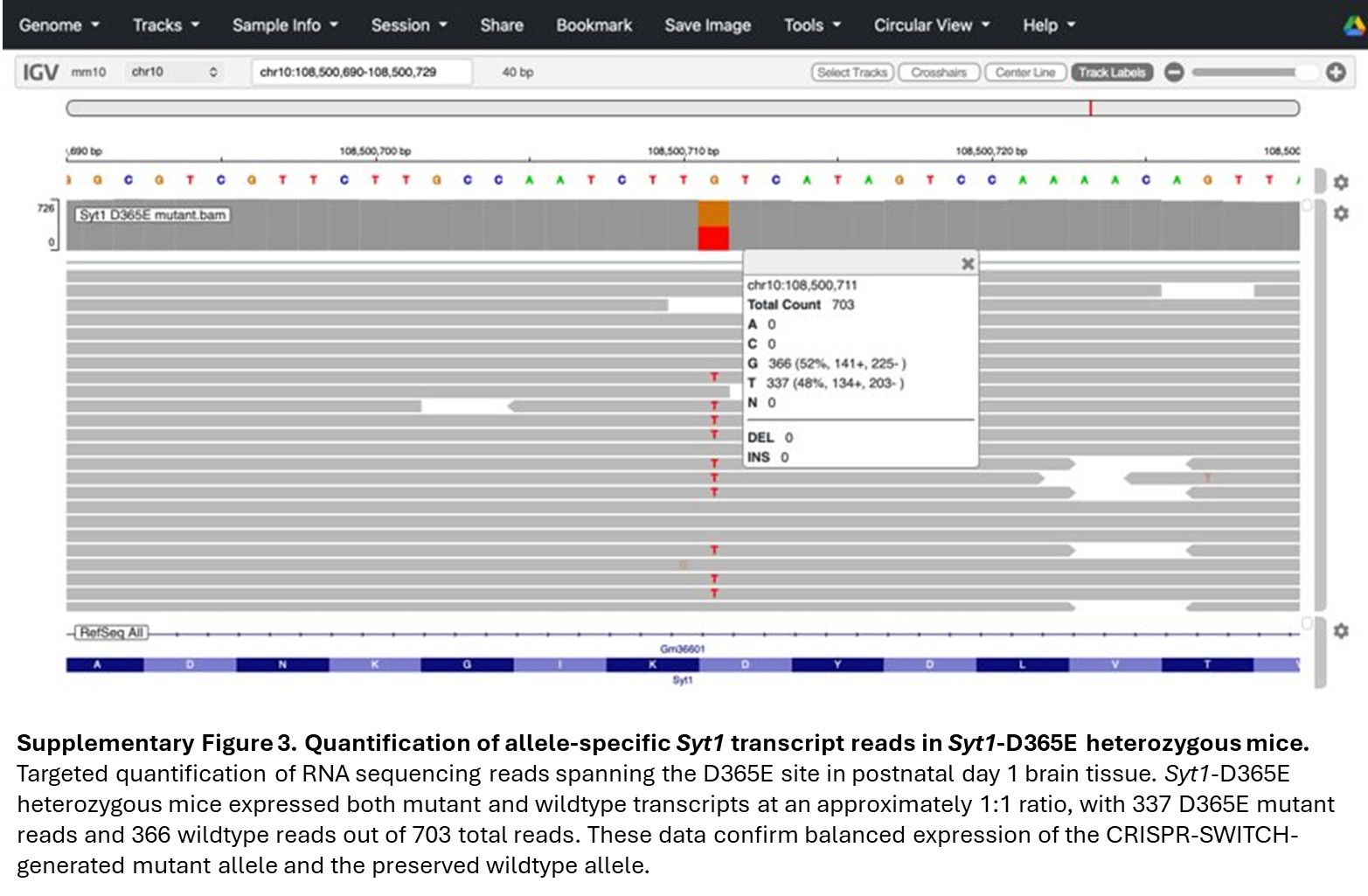

Supplement: Supplementary file 3 [file Image4.jpeg]

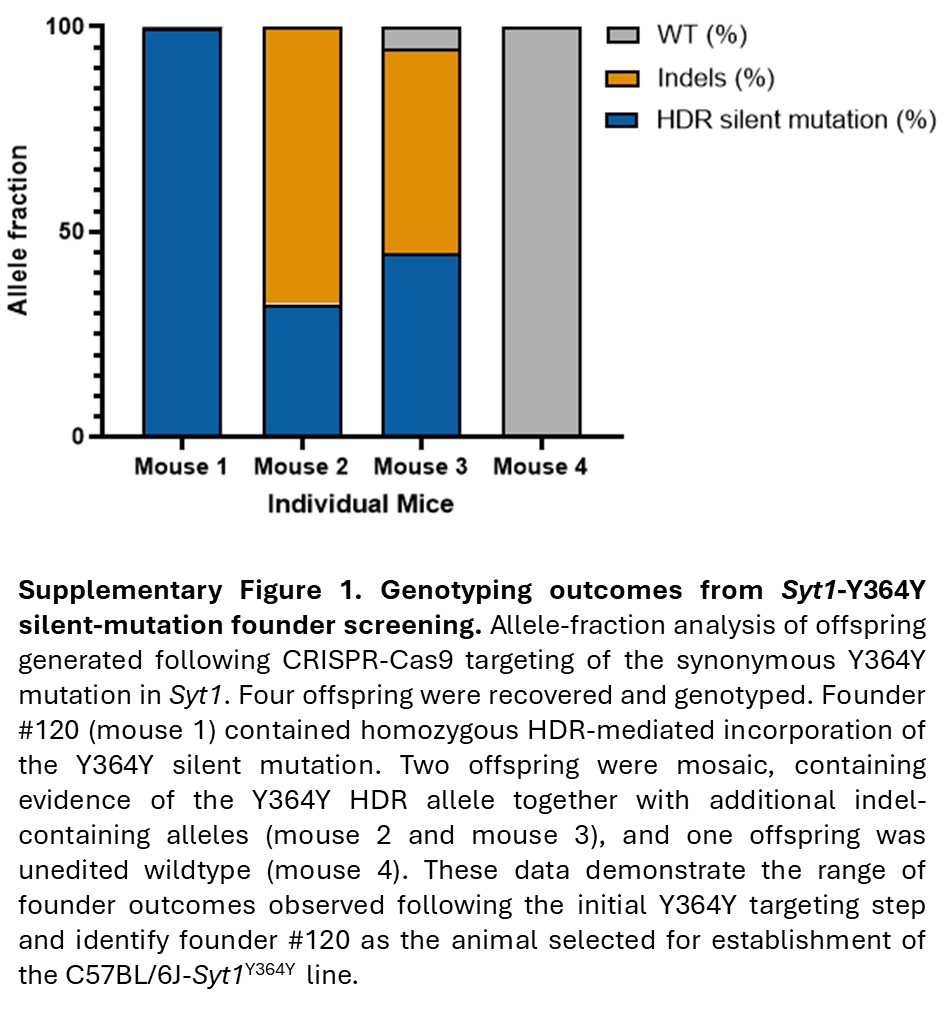

Supplement: Supplementary file 4 [file Image2.jpeg]
